# What neuro-otology specialists need for better care of dizzy patients: a national survey

**DOI:** 10.3389/fneur.2023.1322471

**Published:** 2024-01-08

**Authors:** Georgios Mantokoudis, Andreas Zwergal, Dik Heg, Hassen Kerkeni, Suzie Diener, Roger Kalla, Athanasia Korda, Claudia Candreia, Antje Welge-Lüssen, Alexander Andrea Tarnutzer

**Affiliations:** ^1^Department of Otorhinolaryngology, Head and Neck Surgery, Lnselspital, Bern University Hospital, University of Bern, Bern, Switzerland; ^2^German Center for Vertigo and Balance Disorders (DSGZ), LMU University Hospital, Munich, Germany; ^3^Department of Neurology, LMU University Hospital, Munich, Germany; ^4^CTU Bern, University of Bern, Bern, Switzerland; ^5^Department of Neurology, Lnselspital, Bern University Hospital, University of Bern, Bern, Switzerland; ^6^Practice Neurology St. Gallen, St. Gallen, Switzerland; ^7^Department of Otorhinolaryngology, Head and Neck Surgery, Cantonal Hospital Lucerne, Lucerne, Switzerland; ^8^Department of Otorhinolaryngology, Head and Neck Surgery, University Hospital Basel, Basel, Switzerland; ^9^Neurology, Cantonal Hospital of Baden, Baden, Switzerland; ^10^Faculty of Medicine, University of Zurich, Zurich, Switzerland

**Keywords:** vertigo, dizziness, survey, bedside examination, specialists, diagnosis

## Abstract

**Background:**

A substantial fraction of dizzy patients are assessed by neurologists and ear–nose–throat (ENT) physicians. With the differential diagnosis being broad and often different specialties involved, we aimed to assess the interaction with generalists from the specialists’ perspective to identify limitations and needs and to define strategies for improvement in patient care and education by the specialist.

**Methods:**

One hundred eleven board-certified neurologists (*n* = 62) and ENT physicians (*n* = 49) working in Switzerland participated in an online survey. Here, we focused on limitations faced in the diagnostic workup and treatment of the dizzy patient and potential strategies to improve the standard of care and the interaction between generalists and specialists. Descriptive statistical analyses were performed. We hypothesized that those specialists applying modern concepts in history-taking and bedside examination techniques reach a specific diagnosis more often and request fewer referrals.

**Results:**

Specialists indicated higher confidence in reaching a specific diagnosis for patients presenting with acute dizziness than episodic/chronic dizziness (80% vs. 60%) at the first consultation. Knowledge of the timing-and-trigger concept [odds ratio (OR) = 0.81 (0.67–0.98), *p* = 0.034], as well as of subtle oculomotor/vestibular signs [OR = 0.80 (0.68–0.94), *p* = 0.007] was predictive of the self-reported probability of reaching a specific diagnosis in patients with episodic/chronic dizziness, while no such differences were observed in the care of acutely dizzy patients. Further referrals of acutely dizzy patients were significantly higher in neurologists than in ENT physicians (17% vs. 10%, *p* < 0.001) and in specialists located in the Latin part of Switzerland [OR = 2.84 (1.63–4.93), *p* < 0.001], while this was not the case for patients with episodic/chronic dizziness. Identified unmet needs included regular communication between physicians (27%/53%; always/often true) and sufficiently detailed information on the previous workup from the referrals (27%/53%). Specialists expressed most interest in hands-on courses/workshops, webinars, and practical guidelines for education.

**Conclusion:**

In our survey, bedside state-of-the-art assessments were key in reducing the fraction of unclear dizzy cases. Several gaps were identified that should be addressed. Specifically, referring physicians should provide more comprehensive details regarding urgency, prior diagnostics, and treatment. Specifically, when promoting the knowledge of neurologists and ENT physicians, this should be preferentially done by offering a combination of hands-on courses and webinars.

## 1 Introduction

A relevant fraction of dizzy patients presenting to ambulatory care clinics are assessed by specialists from either ear–nose–throat (ENT) (13.3%) or neurology (9.6%) (1). Similarly, in many emergency departments (ED) dizzy patients are seen by a neurologist or an ENT physician, reaching fractions of up to 35.3 and 11.4%, respectively (2). In a multidisciplinary setting, the rate of vestibular symptoms remaining of unknown origin may be markedly reduced compared to a single specialty being involved (14.3% vs. 20–30%) (3–5). This emphasizes the importance of triage and the value of a specialized assessment in selected cases with acute dizziness. In patients assessed by specialized tertiary dizzy clinics frequently, a change in diagnosis can be observed (6, 7). Specifically, the fraction of undiagnosed cases decreased from 70 to 10% in a Swiss Academic Vertigo Center (6). Similarly, in a South Korean referral-based dizziness clinic run by neurologists, only 5% of patients did not receive a specific diagnosis (8).

Based on a survey performed by primary care physicians (PCPs) in Switzerland, we identified several significant limitations in the care of dizzy patients (9, 10). This included high rates of unclear cases after initial diagnostic assessment and high referral rates to specialists. Furthermore, we found a need for improvement in the communication between referring physicians and specialists. These findings underline the impact of the specialist and the need for appropriate patient journeys in the assessment of the dizzy patient.

Noteworthy, training for neuro-otology during either neurology residency or ENT residency is limited and substantially depends on the expertise of the teaching hospital. Therefore, identifying limitations and unmet needs in the care of dizzy patients by specialists is important. To improve our understanding of the role of specialists (either in private practice or in hospitals) in the care of dizzy patients, we provided a structured questionnaire to board-certified neurologists and ENT physicians. This questionnaire addressed the same aspects as the questionnaire previously used in PCPs, but now from the specialists’ perspective. Again, this questionnaire was designed and used in the context of the Swiss healthcare system (10). Notably, similar challenges are expected for other healthcare systems worldwide. Here, we report on limitations and unmet needs in the care of the dizzy patient and potential targets of specialists’ educational approaches to improve this situation. We hypothesized that familiarity with current diagnostic guidelines and targeted bedside oculomotor examinations (e.g., looking for subtle oculomotor signs), years of professional experience, and the specialty involved would significantly affect the approaches taken to diagnose the cause of dizziness in these patients. Thus, we anticipate that these parameters affect the fraction of cases where this cause remains unclear, which would in turn increase the rate of referral to other specialists and decrease the overall satisfaction. We predicted lower rates of unclear cases and therefore fewer referrals by those specialists being more experienced and familiar with focused individual patient’s history assessments and targeted bedside oculomotor examinations. Furthermore, we also predicted significant differences in reported referral rates, limitations, and unmet needs among different specialties reflecting current perceived knowledge gaps and limitations in knowledge transfer. Based on this survey we will be able to identify existing gaps in the care of dizzy patients by ENT specialists and neurologists and to define key steps for educational strategies (both specialists and patients), eventually reducing delays in diagnosis and treatment and optimizing healthcare resources.

## 2 Materials and methods

### 2.1 Design and delivery of the questionnaire, and identification of suitable participants

For this survey-based study, a structured anonymous online questionnaire was designed by the authors based on their clinical expertise (AZ, GM, and AAT), current guidelines in diagnosing and treating dizzy patients, and limitations previously reported in the literature. The target population of this survey were board-certified neurologists and ENT physicians working in private practice or (academic/non-academic) hospitals in Switzerland, referred to as “specialists” in this manuscript. For the specialists, we used a slightly modified version of the questionnaire originally developed for the survey of primary care physicians (9, 10). Three main sections of the questionnaire were defined to address the pre-specified key aims of the study. While the first section focused on the current situation in the assessment of dizzy patients by specialists, the second section addressed the limitations faced by the specialist in the diagnostic workup and the treatment of the dizzy patient. In the third section, potential strategies to improve the standard of care of the dizzy patient and the interaction between generalists and specialists were discussed, and the value of different teaching formats was evaluated (see Appendix for the full questionnaire). The estimated time needed to fill out the questionnaire was 20–25 min. The questionnaire was available in both German and French language, and the translation from German to French was supervised by a native French-speaking expert in the field. No validation of this survey was performed. Survey Monkey (Momentive Global Inc., San Mateo, CA, United States) was used for the delivery of this online-only questionnaire. Invitations for participation were sent to board-certified neurologists and ENT physicians who had previously agreed to be registered in a medical database run by healthbook.ch.

### 2.2 Statistical analysis of the questionnaire

First, a descriptive statistical analysis of the questionnaire was performed, focusing on epidemiologic aspects including office size and location, years of professional experience, and specialty. Second, univariable and multivariable statistical analyses were run to validate the pre-specified hypotheses. Statistical support was provided by DH from the clinical trial unit (CTU) of the University of Bern (Switzerland).

A series of scores to reflect key aspects of the diagnostic workup (both history taking and bedside testing) were predefined by the authors (AZ, GM, and AAT). These scores were graded based on the extent to which the specialists agreed with a given procedure or the indicated importance of a proposed measure used (see Supplementary Table S1 in the Appendix for detailed descriptions of the scores used and how they were calculated). Overall scores were derived from the summed items and then indexed to 0–100% if items were measured on the same scale. Otherwise, items were first normalized to 0–100% and then averaged to retrieve the overall score. Fractional regressions [odds ratio (OR) with 95% confidence intervals (CIs)] are reported for indexed scores (0 to 100%); otherwise, binary dependent variables were analyzed with logistic regressions (OR with 95% CI). These scores were then correlated with epidemiologic aspects such as years of professional experience, location of the specialists’ office, and reported number of dizzy patients evaluated. No validation of these scores was performed.

All statistical analyses were performed using Stata version 17. Descriptive statistics report means with standard deviations (±SD), medians with inter-quartiles (25 to 75%), or counts with percentages (% of non-missing cases) and sample sizes (number of respondents).

## 3 Results

We contacted 959 neurologists and 373 ENT physicians. A total of 111 completed surveys from either board-certified ENT physicians (*n* = 49; return rate = 13.1%) or neurologists (*n* = 62; return rate = 6.5%) were included. The majority of participants were men (64%), worked in private practice (56%), and aged 41–60 years (55%). Notably, in the neurology group, a significantly higher fraction of younger (aged 40 years or less) participants were identified than in the ENT physicians’ group (40% vs. 8%, *p* < 0.001). Similarly, the fraction of participating specialists working in hospitals were substantially larger for neurologists than for ENT physicians (66% vs. 16%). For those specialists working in private practice (*n* = 62), offices were located in cities in most cases (69%), whereas offices in the agglomeration (9%) or rural offices (8%) were less frequent. On average, (±1SD) participating specialists saw 14.7 ± 7.9 patients per day, spending 24.4 ± 6.5 min per patient. Importantly, ENT physicians reported seeing a significantly higher number of patients per day than neurologists (21.0 ± 7.6 vs. 9.8 ± 3.4), resulting in lower consultation times (20.5 ± 5.9 min vs. 27.4 ± 5.3). Over the period of a month, the number of patients seen with a leading symptom of dizziness averaged 19.3 (±15.9, 1SD, range: 0–100 patients), with numbers for ENT physicians (20.7 ± 19.0) and neurologists (18.1 ± 13.0) not being significantly different (*p* > 0.05).

### 3.1 Diagnostic limitations reported by specialists when taking care of dizzy patients

#### 3.1.1 Unclear diagnoses and self-confidence in the diagnostic workup and treatments initiated

**Figure 1:**
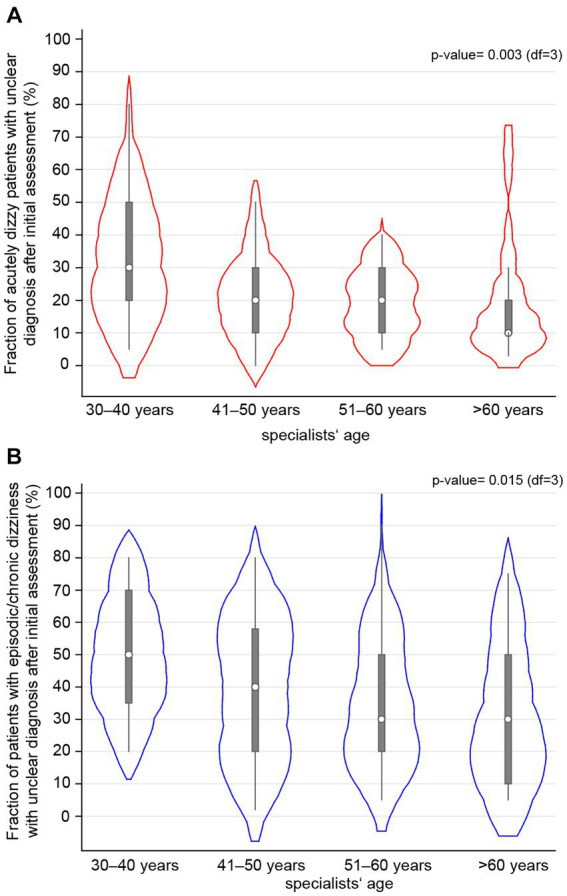
The fraction of dizzy patients who did not receive a specific diagnosis after the initial assessment by the specialists is correlated with the specialists’ age, with results shown separately for patients with acute **(A)** or episodic/chronic **(B)** dizziness using a violin plot. All specialists were assigned to one of four age bins. The white circle represents the median value, the error bars provide the inter-quartile range (with the lower edge of the bar indicating the 25% percentile, the upper end of the bar indicating the 75% percentile, and the thin lines indicating the lower and upper adjacent values), and the red **(A)** or blue **(B)** cloud represents the distribution of all the specialists of that age group.

Specialists indicated that only in a minority of cases, no specific diagnosis could be reached after the first consultation in patients with acute dizziness [20% (10; 30)], whereas rates were substantially higher for patients with episodic/chronic dizziness [40% (20, 55)] (see Table 1). When performing logistic regression analyses with regards to the odds of lacking a specific diagnosis after the initial assessment of acutely dizzy patients using various epidemiologic parameters and several scores (see Supplementary Table S2), only age (*p* = 0.003) and the location of the specialist’s office (German vs. Latin part of Switzerland) had a significant impact. Specifically, the fraction of acutely dizzy patients with unclear diagnosis after initial assessment was significantly higher for specialists working in the Latin part of Switzerland [OR = 1.61 (1.11–2.33), *p* = 0.012], whereas it was inversely related to the years of professional experience [OR = 0.78 (0.64–0.94), *p* = 0.008]. Furthermore, those specialists aged 30–40 years reported significantly increased odds [OR = 2.03 (1.15–3.59), *p* = 0.015] for reaching no specific initial diagnosis in acutely dizzy patients after the first consultation, compared to those specialists aged more than 60 years (see Figure 1A). However, this was not confirmed in a multivariable analysis, suggesting other mediating factors (i.e., the other factors used in the multivariable model: location of the specialist’s office, professional experience, number of dizzy patients seen per month, “timing & triggers” score, “subtle oculomotor and vestibular signs” score, and “superscore acute vertigo/dizziness”) may reduce this self-reported gap in reaching a satisfactory initial diagnosis (*p* = 0.265).

**Table 1:** Referral patterns and lack of specific diagnosis (ENT, neurology).

Specialists considered for further evaluation of the dizzy patient (in order of decreasing frequency) (*n* = 111)	
Emergency physicians	1.0 [1.0; 3.0]
Ear-nose-throat (ENT) physicians	3.0 [2.0; 5.0]
Neurologists	3.0 [2.0; 7.0]
Cardiologists	4.0 [3.0; 5.0]
Psychiatrists	5.0 [4.0; 6.0]
Interdisciplinary center for assessing vertigo/balance disorders	5.0 [4.0; 7.0]
Neurosurgeons	6.0 [4.0; 7.0]
Spinal cord surgeons	7.0 [6.0; 8.0]
Lack of specific diagnosis (*n* = 111)	% (mean [IQR])
*In patients with acute dizziness*	
After the first consultation	20% [10%; 30%]
Upon completion of the diagnostic workup	10% [5%; 20%]
*In patients with episodic/chronic dizziness*	
After the first consultation	40% [20%; 55%]
Upon completion of the diagnostic workup	20% [10%; 40%]

Specialists considered median rank and IQR ranks in brackets [25%; 75%]. 1 = first specialist considered, 8 = last specialist considered. Lack of specific diagnosis: % reported by the specialists, median [25 and 75% interquartiles].

When applying logistic regression analyses focusing on patients presenting with episodic or chronic dizziness using various epidemiologic parameters and several scores (see Supplementary Table S3), again, age (*p* = 0.015) had a significant impact on the self-reported fraction of patients receiving a specific initial diagnosis. Specifically, those specialists aged 30–40 years demonstrated significantly increased odds [OR = 1.98 (1.19–3.31), *p* = 0.009] for reaching no specific diagnosis in patients with episodic/chronic dizziness after the first consultation compared to those specialists aged more than 60 years (see Figure 1B). This, however, was again not confirmed in a multivariable analysis (*p* = 0.409), probably due to other compensatory factors playing a role.

The fraction of patients with episodic/chronic dizziness with unclear diagnosis after initial assessment was significantly higher for specialists working in the Latin part of Switzerland [OR = 1.61 (1.10–2.36), *p* = 0.015]. Years of professional experience [OR = 0.80 (0.68–0.95), *p* = 0.009], knowledge of the “timing & triggers” score [OR = 0.81 (0.67–0.98), *p* = 0.034], the “subtle oculomotor and vestibular signs” score [OR = 0.80 (0.68–0.94), *p* = 0.007], the “essential” in episodic/chronic dizziness score [OR = 0.74 (0.62–0.88), *p* < 0.001], and the “superscore for episodic/chronic dizziness” [OR = 0.64 (0.51–0.80), *p* < 0.001] were inversely related to the probability of reaching no specific diagnosis in patients with episodic/chronic dizziness after initial assessment (see Supplementary Table S3). Notably, when using a multivariable logistic regression instead, none of these scores emerged as a strong predictor.

A majority of specialists indicated that they often or always felt confident in their assessment and treatment of the patient with acute (97%/93%) or episodic/chronic (90%/83%) vertigo or dizziness and that they were at least often satisfied with the results of the diagnostic workup performed (92%/81%, acute/episodic or chronic dizziness) (see Supplementary Figure S1 for details).

#### 3.1.2 Referral patterns of specialists for dizzy patients and triggers for further evaluation

**Figure 2:**
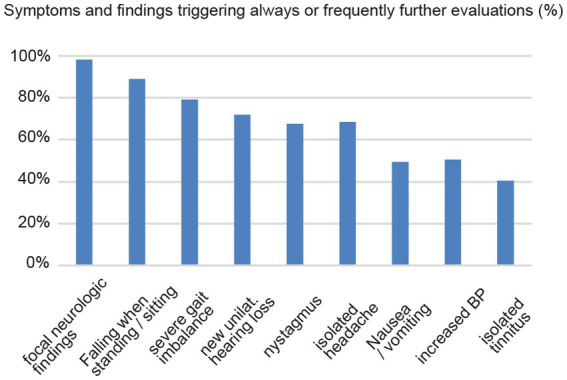
Board-certified ENT (ear-nose-throat) physicians and neurologists indicated in which percentage of cases various symptoms and findings will always or frequently trigger further evaluation.

Specialists indicated that 10% [5.0; 25.0] (median [IQR]) of all acutely dizzy patients and 10% [5.0; 30.0] of all patients with episodic or chronic dizziness were sent to other specialists for further evaluation, with fraction of reported referrals in case of acutely dizzy patients being significantly higher for neurologists than for ENT physicians [17% (10.0; 33.3) vs. 10% (2.0; 10.0), *p* < 0.001]. For further referral, specialists most frequently considered sending their patients to the ED [ranking: 1.0 (1.0; 3.0)], to (other) ENT physicians [ranking: 3.0 (2.0; 5.0)], and to (other) neurologists [ranking: 3.0 (2.0; 7.0)] (see Table 1 for details). In patients with acute dizziness, participating specialists agreed that the presence of various symptoms or findings will always or frequently trigger further evaluation, as illustrated in Figure 2.

When performing a univariable regression analysis with regards to the odds of referring an acutely dizzy patient to another specialist or the ED, the location of the interviewed specialist’s working place, years of professional experience, and an unclear diagnosis after the specialist’s initial assessment showed significant effects. Specifically, specialists with an office in the Latin part of Switzerland [OR = 2.84 (1.63–4.93), *p* < 0.001], those specialists working in a hospital [OR = 3.53 (1.74–7.18), *p* < 0.001], and those with higher fractions of unclear diagnoses [OR = 1.37 (1.22–1.54), *p* < 0.001] made significantly more referrals to other specialists or to the ED. In contrast, the likelihood of referral decreased with increasing years of professional experience [OR = 0.78 (0.61–1.00), *p* = 0.048]. This was confirmed in a logistic regression analysis (see Supplementary Table S4).

A small majority of participating neurologists indicated that it was always (19%) or often (33%) true that patients with episodic/chronic dizziness are sent for further evaluation and treatment to an ENT specialist or to another neurologist. In contrast, only a minority of participating ENT physicians indicated that it was always (5%) or often (20%) true that they send such patients to other ENT physicians or neurologists for further evaluation. For patients with episodic or chronic dizziness, the location of the interviewed specialist’s working place, years of professional experience, and an unclear diagnosis after the specialist’s initial assessment showed significant effects on the odds of referrals to another specialist. Specialists with an office in the Latin part of Switzerland [OR = 2.61 (1.47–4.63), *p* = 0.001], those specialists working in the agglomeration, in the city [OR = 2.65 (1.24–5.67), *p* = 0.012], or a hospital [OR = 3.47 (1.84–6.57), *p* < 0.001], and those with higher fractions of unclear diagnoses [OR = 1.20 (91.06–1.35), *p* = 0.004] made significantly more referrals to other specialists. This was confirmed in a multivariable analysis, but only for the fraction of unclear diagnoses (see Supplementary Table S4). Notably, the likelihood of referral of patients with episodic/chronic dizziness did not strongly depend on the years of professional experience in either univariable or multivariable analyses.

### 3.2 Unmet needs identified by the specialists and ways to improve the care of the dizzy patient

#### 3.2.1 Interaction between interviewed specialists and referring physicians

When evaluating dizzy patients referred from either primary care physicians (PCPs) or other specialists, a majority of participating specialists agreed that they would like to see an improved dialogue between the referring physician and the specialist (20%/45%; always/often true) and that they would like to receive more detailed information about the urgency of the referral (19%/44%) and the previous diagnostic workup and treatment (27%53%) (see Supplementary Figure S2 for details). In total, 45% of specialists indicated that they would like to see the referring physician consistently take back patients for further treatment (11%/34%). With regards to the threshold of referral, more specialists indicated that they would like to see more selective referrals (19%/49%) than faster referrals in the case of unclear presentation (17%/39%).

#### 3.2.2 Approaches to improve the specialists’ knowledge about vertigo and dizziness

Among different strategies proposed, participating specialists considered hands-on courses and workshops (46%/36%; always/often true), webinars (32%/44%), and national recommendations/printed guidance papers (37%/38%) most often suitable to improve their knowledge about dizziness (see Table 2 and Supplementary Figure S3 for details). Practical (printed) recommendations (29%/43%) were also considered suitable to improve their skills in taking care of dizzy patients by a majority of specialists, whereas smartphone apps for teaching and providing recommendations were slightly less popular (23%/25%).

**Table 2:** Tools to improve the management of the dizzy patient^a^.

Tools considered helpful in the diagnosis/treatment	Fractions (%) of agreement (diagnosis/treatment)
Digital pathways/algorithms (web-based)	75/111 (68%) / 78/111 (70%)
Digital pathways/algorithms (smartphone App)	52/111 (47%) / 61/111 (55%)
Web portal with clinical cases	72/111 (65%) / 61/111 (55%)
Other	3/111 (3%)^b^ / 3/111 (3%)^c^

Tools considered helpful for patient education	Fractions (%) of agreement
Brochure for patients (print)	85/111 (77%)
Flyer for patients (print)	71/111 (64%)
Digital platform (app-based)	64/111 (58%)
Dizzy diary (print)	58/111 (52%)
Digital platform (web-based)	55/111 (50%)
Other	1/111 (1%)^d^

Tools considered helpful in the follow-up	Fractions (%) of agreement
App for follow-up including a dizzy diary (digital)	68 /111 (61%)
Dizzy diary (print)	66 /111 (59%)
Web-based follow-up (digital)	47 /111 (42%)
Web portal with clinical cases	40 /111 (36%)
Other^a^	2 /111 (2%)^e^

a Note that there were no significant differences between neurologists and ENT physicians, thus results were pooled.

b Other approaches considered helpful for improving the diagnostic workup mentioned were literature research (*n* = 1), watching teaching videos (*n* = 1), and continuous medical education (*n* = 1).

c Other approaches considered helpful for improving the treatment mentioned were reading guidelines (*n* = 1), watching teaching videos (*n* = 1), and continuous medical education (*n* = 1).

d Other follow-up strategies considered helpful were vestibular event monitoring by the patient (*n* = 1) and using a suitable smartphone app (*n* = 1).

e Other educational tools considered helpful mentioned were instructions for self-treatment provided by a pharmaceutical company (*n* = 1).

#### 3.2.3 Approaches to improve the care of dizzy patients

Among different digital approaches proposed, participating specialists most often considered web-based digital pathways and algorithms helpful in the diagnostic workup (68%) and when treating (70%) dizzy patients. Rates for agreement for other digital tools (app-based digital pathways and algorithms and web portals providing clinical cases) were slightly lower, see Table 2 for details. For following up on dizzy patients, apps including a digital dizzy diary (61%) and the use of a printed dizzy diary (59%) were considered helpful by the largest fraction of specialists. With regards to different educational strategies proposed, printed brochures for patients (77%) were considered most helpful by participating specialists, followed by printed flyers for patients (64%) and app-based digital platforms (55%).

## 4 Discussion

In this publication, we focused on current limitations, unmet needs, and strategies to overcome difficulties faced by neurologists and ENT physicians involved in the diagnosis and subsequent care of the dizzy patient. We have previously addressed these aspects using a very similar questionnaire from the perspective of primary care physicians (PCPs) (9, 10). By identifying these limitations in both primary care physicians and specialists and by proposing tools to improve their interactions, we aim to develop strategies to enhance the care of dizzy patients overall.

### 4.1 Diagnostic limitations and referral challenges

Specialists indicated that in 40% of patients presenting with episodic or chronic dizziness, no specific diagnosis could be reached after the first consultation (with half of these patients still lacking a specific diagnosis after completion of the diagnostic workup). Nevertheless, the vast majority of specialists frequently (or always) felt confident in assessing and treating patients with episodic/chronic dizziness. Knowledge and use of guideline-supported diagnostic tools suitable for the assessment of these types of dizziness may alleviate this apparent discrepancy. Probably not surprisingly, rates for specialists being satisfied at least often (if not always) with the results of the assessments initiated were slightly higher in acutely dizzy patients (92%) than in patients with episodic or chronic dizziness (81%). We hypothesized that being more familiar with key elements of history taking and bedside examination (such as the HINTS(+) (11, 12), see also our combined “scores” or “superscores”) would result in lower fractions of unclear dizzy cases. Logistic regression analyses that were performed demonstrated such a dependency, with the “timing&triggers” score [referring to the TiTrATE approach (13)], the “subtle oculomotor and vestibular signs” score (14), the “essential” in episodic/chronic dizziness score, and the “superscore for episodic/chronic dizziness” being inversely related with the probability of reaching no specific diagnosis in patients with episodic/chronic dizziness after initial assessment. Furthermore, years of professional experience were inversely related to the likelihood of not reaching a specific diagnosis both for patients with acute and episodic/chronic dizziness. This finding is consistent with the higher rate of unclear cases after assessment by specialists aged 40 years or younger and emphasizes the value of professional experience when taking care of dizzy patients. As an alternative hypothesis, the impact of specialists’ age on the rate of unclear cases could be related to the required level of confidentiality for a given working diagnosis. Thus, we speculated whether younger specialists could require more rigid diagnostic criteria before an unclear case becomes a specific diagnosis. Either way, this finding underlines that when providing teaching activities to specialists, age should be taken into account.

The observed regional differences in the likelihood of reaching a specific diagnosis in dizzy patients, with significantly higher odds of not reaching a specific diagnosis for specialists working in the Latin part of Switzerland, were unanticipated and need to be further studied. At this time, we can only speculate about potential reasons for this finding. Possibly, differences in the diagnostic approach learned and the philosophy taught to address the dizzy patient (coming from different “schools” due to their spoken language) or regional differences in patient’s preferences (e.g., preferring local workups and no referrals and fewer tests) may explain these findings.

The interaction between the referring physician and the specialists was reported as a key element: e.g., improving the dialog between the referring physician and the specialist, receiving more detailed information about the urgency of the referral and the previous diagnostic workup and treatments were those requirements most frequently mentioned. In general, a majority of specialists indicated that they would like to see faster, but more selective referrals of unclear cases. In one study ENT physicians indicated that approximately 30% of audio-vestibular referrals (including non-otologic dizziness) received were considered unnecessary, resulting in a loss of productivity and time (15). While various causes could lead to delayed referral to the specialist (including both patient’s delay and doctor’s delay), reducing the number of patients with no clear diagnosis after the initial assessment of the referring physician will be essential. For PCPs, who provide the initial assessment of the majority of dizzy patients (1), we have discussed potential strategies to address this limitation in a previous publication (9). With regards to the continuation of care of the dizzy patient (either by the specialist or the referring physician), approximately half of the specialists indicated that they would like to see the referring physician consistently take back patients for further treatment. Thus, there seems to be no clear preference with regard to follow-up visits. Obviously, a lack of expertise or time of the referring physician may limit his/her ability to take back the patient. However, with more follow-up consultations performed by specialists, the capacity to see new patients will be more limited. Notably, we previously reported a preference for PCPs to take back patients after the specialists’ assessment for further management (10), which seems to match the expectations expressed by specialists.

Additionally, an unselective triage of all dizzy patients to ENT physicians has been previously identified by others (16), instead of more appropriate referrals to different physicians based on the presenting symptoms. Intensifying the dialogue between specialists and referring physicians may address some of these limitations, as previously discussed by others (17). Furthermore, interprofessional management may also help in more targeted referrals to the most suitable specialist (16).

### 4.2 Different referral patterns by interviewed neurologists and ENT physicians

We noted substantially different referral patterns of dizzy patients reported by specialists included. Overall, neurologists tended to send both patients with acute unilateral vestibulopathy (54% vs. 5%, neurologists vs. ENT physicians) and patients with episodic or chronic dizziness (52% vs. 25%) more often to other specialists (either neurologists or ENT physicians) than ENT physicians. Accordingly, neurologists considered referrals to the ED more frequently than ENT physicians (21% vs. 6%). Similarly, referrals of patients with episodic or chronic dizziness to an interdisciplinary dizzy clinic were indicated significantly more often by neurologists than by ENT physicians (52% vs. 19%). Possibly, lower referral rates by ENT specialists than by neurologists could be related to the necessity to assess hearing function in these patients. While virtually all ENT physicians have access to pure tone audiometry, this is much less often the case for neurologists.

Interestingly, the workplace had a substantial impact, with those specialists working in the Latin part of Switzerland and/or working in a hospital showing significantly higher referral rates for patients with either acute or episodic/chronic dizziness. For acutely dizzy patients, the years of professional experience (with lower odds of referral with more extensive professional experience) had a significant impact, whereas, for patients with episodic/chronic dizziness, the likelihood of further referral was inversely correlated with the knowledge of the “superscore” for episodic/chronic dizziness and the number of dizzy patients seen per month. While we can only speculate about the reasons for such regional differences in the referral pattern of dizzy patients (potentially related to the patient’s preference for further diagnostic workup, or low-threshold accessibility to nearby specialists), higher rates of referrals indicated by specialists working in hospitals are most likely linked to the availability of other specialists nearby.

While neurologists indicated further referral of dizzy patients substantially more often than ENT physicians, both specialties reported very similar rates of self-confidence in the diagnostic workup performed and the treatment initiated. Potential explanations for these differences in the referral pattern may be related to a referral bias, with ENT specialists being more likely to assess patients with obvious peripheral-type patterns and neurologists being asked to further evaluate patients with more subtle or unspecific symptoms and findings. Such differences in the patient populations seen by these two specialties were also reflected in the ranking of diagnoses most frequently made, with acute unilateral vestibulopathy and Menière’s disease receiving higher rankings by ENT physicians than by neurologists and conversely, dizziness and gait imbalance related to peripheral neuropathy being more frequently seen by neurologists than by ENT physicians. Despite this high level of self-confidence, all specialists reported substantial rates of unclear diagnoses of episodic/chronic dizziness after their initial diagnosis.

With regards to referrals to the ED for further diagnosis and treatment, this could be related to the underlying working diagnosis by the referring physician, sending patients with suspected central (ischemic) causes preferentially to neurologists. In these cases, further diagnostic workup is usually performed in the ED and in the stroke unit, whereas an acute unilateral vestibulopathy is more likely to be managed in an outpatient setting. Importantly, diagnostic equipment for more detailed and quantitative testing of peripheral-vestibular function is more frequently available to ENT physicians than to neurologists. Based on vestibular function testing, the previous diagnosis was revised in 54% in a single study, emphasizing its impact on the diagnostic workup (18). Thus, ENT physicians will be less likely to refer patients further for quantitative audio-vestibular testing. With a significantly larger fraction of neurologists working in hospitals than ENT physicians (32% vs. 8%) in this survey, this could have facilitated further referrals for neurologists (having access to both the ED and other specialists in-house).

### 4.3 Enhancing vertigo and dizziness care by educational tools

Based on the specialists’ feedback on preferred educational approaches, face-to-face teaching such as hands-on courses or workshops, webinars, and national recommendations/printed guidance papers should be prioritized. The lower levels of priority for other digital content such as smartphone apps may be linked to the demographics of the participating specialists. With webinars receiving almost the same level of acceptance while being less demanding with regard to infrastructure and accessibility as in-person courses, this virtual teaching format should be expanded. However, for improving practical skills (e.g., HINTS testing (11)), in-person practical courses remain the most suitable format and thus should be offered as well. A recent study showed that video instructions for teaching head impulse testing (which is part of the HINTS battery) were not sufficient in order to reach a meaningful testing quality in terms of correct head movements (19). Importantly, gaps of knowledge may vary substantially among different countries and continents, as, e.g., shown for the use of provocation and repositioning maneuvers in suspected posterior canal benign paroxysmal positional vertigo (BPPV). Based on the results from a survey of Lithuanian physicians reporting no use of provocation maneuvers (neurologists = 24%, ENT physicians =33%) and repositioning maneuvers (neurologists = 28%, ENT physicians = 61%) for suspected posterior canal BPPV (20), educational approaches should prioritize diagnosing and treating posterior canal BPPV. In contrast, in our survey both neurologists and ENT physicians were very familiar with posterior canal BPPV treatment, but the neurologists had knowledge gaps for diagnosing and treating lateral canal BPPV (as reported in a companion paper currently under review). Thus, in Switzerland, teaching activities regarding BPPV should focus on neurologists and PCPs (10) and the lateral canal. This emphasizes significant national differences and the importance of adapting teaching activities to the specific needs of a given country.

Reviewing the indicated preferences, web-based digital algorithms and pathways and web portals should be developed and distributed with priority for supporting the specialists in the diagnosis and treatment of the dizzy patient. Similarly, for following up on dizzy patients, the use of a smartphone app for follow-up (including a digital dizzy diary) or a printed dizzy diary was considered most useful by the specialists. Thus, these formats should be prioritized. Regarding educational materials for patients, a preference for printed information material (brochures or flyers) was observed. These findings emphasize the need to continue providing printed information materials to patients, despite the regular use of smartphones by elderly patients.

### 4.4 Study limitations

This study has several limitations that need to be considered. This includes a potential selection bias for participating in this survey based on the physician’s interest in taking care of the dizzy patient and a recall bias of participants. Notably, response rates of suitable specialists invited to participate in this survey were low for ENT physicians (13.1%) and even more so for neurologists (6.5%). Potential reasons for a low return rate include lack of time considering the estimated duration for completion of the survey of 20 to 25 min and lack of expertise in taking care of dizzy patients. The finding that specialists reported substantial rates of unclear diagnoses of episodic/chronic dizziness after their initial diagnosis despite a high level of self-confidence in the care of dizzy patients was puzzling and should be further evaluated. Reducing the rate of unclear cases should be prioritized. Furthermore, the identified unmet needs will also likely be influenced by the epidemiologic differences between the two specialties including the workplace place setting (e.g., office location, multidisciplinary setting, and size) and the years of professional experience. These differences may also have an impact on the preferred tools for education, with younger participants likely having a stronger preference for digital content than more elderly specialists. With regards to the differences in the reported fraction of unclear cases after an initial assessment based on the workplace (Latin vs. German part of Switzerland), the relative underrepresentation of specialists from the Latin part of Switzerland emphasizes the need to further study such regional differences. While we focused on the Swiss healthcare system, we do expect similar needs and gaps in knowledge in specialists taking care of dizzy patients in other countries. Nonetheless, country-specific differences in the design of the healthcare system may influence referral rates, the extent of the diagnostic workup, and suitable educational approaches. Thus, caution is advised when transferring our findings to other healthcare systems.

## 5 Conclusion

Specialists familiar with detailed history taking, timing, triggers, and subtle oculomotor signs during bedside examinations achieve more accurate diagnoses in patients with episodic/chronic vertigo or dizziness, emphasizing the importance of state-of-the-art bedside assessments. The needs of neurologists and ENT physicians have to be addressed separately since we found differences in patient care especially in aspects of referrals, diagnostic equipment, and the assessment of lateral canal BPPV. Referring physicians should provide more comprehensive details regarding urgency, prior diagnostics, and treatment. In addition, the responsible party for patient follow-up should be clearly specified. The findings from this survey will guide the development of tools to improve the diagnosis and treatment of dizzy patients. Specifically, when promoting the knowledge of neurologists and ENT physicians, this should be preferentially done by offering a combination of hands-on and webinars or even comprehensive training programs (e.g., provided at national conferences), whereas for patient education, printed brochures and flyers should be provided by both involved specialists and primary care physicians and patient organizations.

## Data availability statement

The raw data supporting the conclusions of this article will be made available by the authors, without undue reservation.

## Author contributions

GM: Conceptualization, Formal analysis, Methodology, Validation, Writing – review & editing. AZ: Conceptualization, Formal analysis, Methodology, Validation, Writing – review & editing. DH: Data curation, Formal analysis, Methodology, Visualization, Writing – review & editing. HK: Conceptualization, Formal analysis, Methodology, Writing – review & editing. SD: Methodology, Validation, Writing – review & editing. RK: Formal analysis, Methodology, Writing – review & editing. AK: Formal analysis, Methodology, Writing – review & editing. CC: Formal Analysis, Methodology, Writing – review & editing. AW-L: Formal analysis, Methodology, Writing – review & editing. AT: Conceptualization, Data curation, Formal analysis, Investigation, Methodology, Project administration, Resources, Supervision, Validation, Visualization, Writing – original draft, Writing – review & editing.
