# Supplementary material for: What neuro-otology specialists need for better care of dizzy patients: a national survey
Source: Front Neurol. 2024 Jan 8;14:1322471. doi: 10.3389/fneur.2023.1322471 (PMC10800397; doi:10.3389/fneur.2023.1322471)
Supplement: Supplementary file 1 [file Data_Sheet_1.PDF]

What neuro-otology specialists need for a better care of dizzy patients – a national survey

Supplementary materials

FIGURES

Figure S1 Specialists' satisfaction with diagnostic and therapeutic procedures performed in dizzy patients

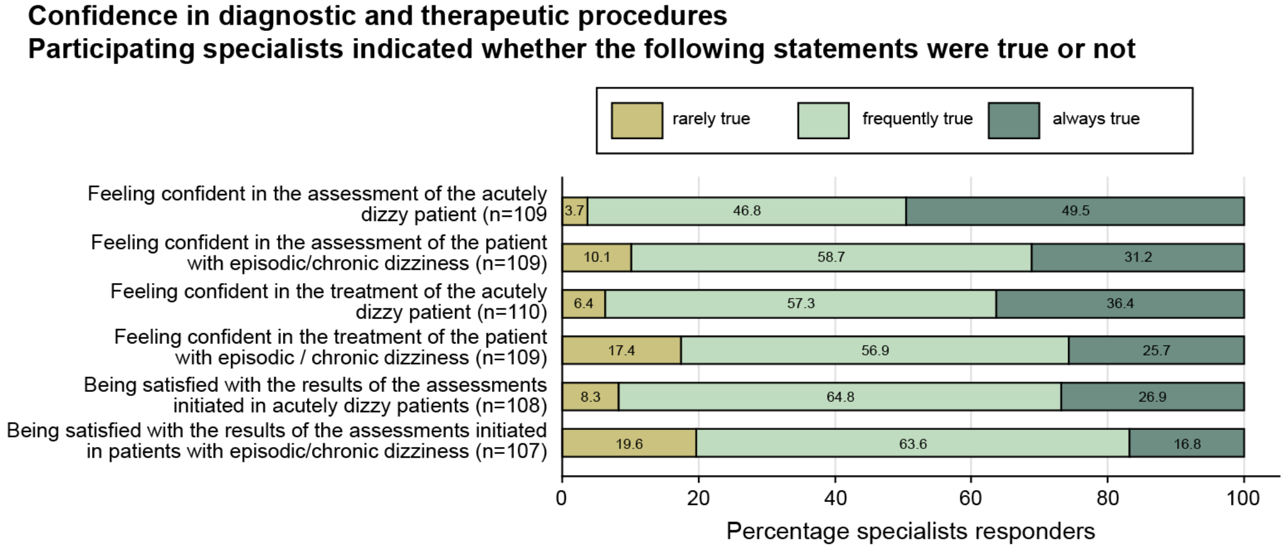

Figure legend:
Response patterns of participating specialists are shown for a series of statements addressing the specialists' satisfaction with diagnostic and therapeutic procedures performed in dizzy patients. For each question, the percentage of specialists and the extent of agreement they indicated (ranging from "never true" to "always true") are illustrated. For each question the number (n) of valid replies are provided in brackets. Note that none of the specialists selected "never true".

Figure S2: specialists’ satisfaction regarding the interaction with the general practitioners

Referrals
Participating specialists indicated whether the following statements were true or not

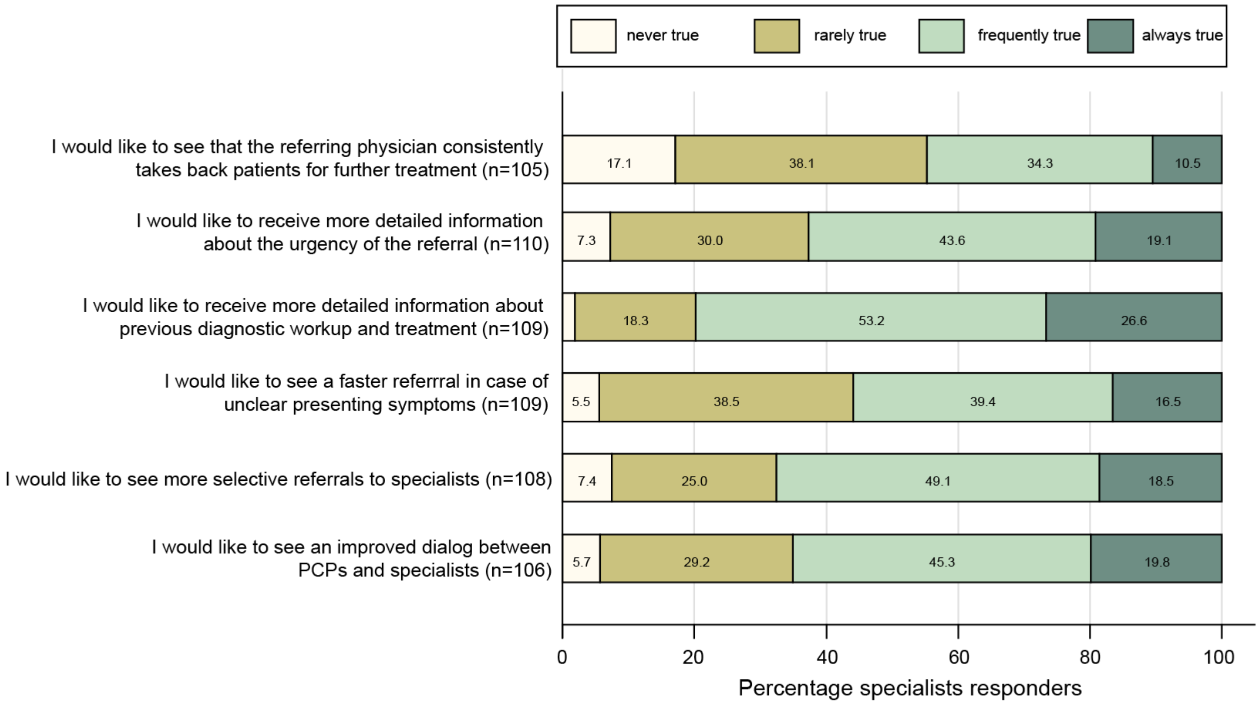

Figure legend:
Response patterns of participating specialists are shown for a series of statements focusing on the specialists’ satisfaction regarding referrals of dizzy patients to specialists. For each question, the percentage of specialists and the extent of agreement they indicated (ranging from “never true” to “always true”) are illustrated. For each question the number (n) of valid replies are provided in brackets.

Figure S3: Approaches to improve the specialists' knowledge about vertigo and dizziness

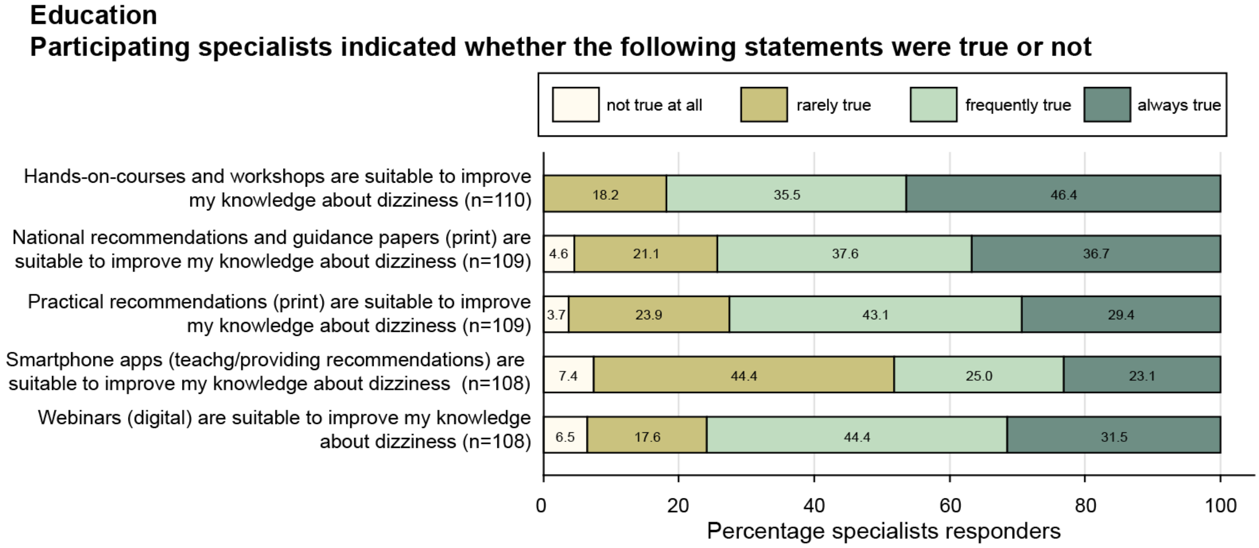

Figure legend:
Response patterns of participating specialists are shown for a series of statements focusing on educational approaches to improve the specialists' knowledge about vertigo and dizziness. For each question, the percentage of specialists and the extent of agreement they indicated (ranging from "never true" to "always true") are illustrated. For each question the number (n) of valid replies are provided in brackets.

TABLES

Table S1: Description of scores used

Table S1: Description of scores used	
**Score**	**Items addressed**
“Timing & triggers” score	Frequency and duration of dizzy spells, triggers (specific body movements / positions, specific situations), accompanying symptoms [1])
HINTS	**H**ead-**I**mpulse test, looking for gaze-evoked **N**ystagmus and **T**est of **S**kew (looking for a skew deviation [2]
HINTS+	HINTS score plus looking for new-onset unilateral hearing loss [3]
“Hearing” score	Testing for new-onset hearing loss, performing an otoscopy
“Ataxia of stance and gait” score	Walking on the line (with/without viewing), Romberg test, Unterberger stepping test
“Subtle oculomotor and vestibular signs” score	Performing HINTS and testing for spontaneous nystagmus with both fixation preserved and removed
“Essential in acute vertigo / dizziness” score	Testing for HINTS+, assessment of walking on the line (with/without viewing), Romberg test and for spontaneous nystagmus with both fixation preserved and removed
“Essential in episodic / chronic vertigo / dizziness” score	Performing provocation maneuvers, the head-impulse test, assessments of walking on the line (with/without viewing) and the Romberg test
“Essential in suspected BPPV” score	Asking for timing & triggers [1] and performing provocation maneuvers
“Superscore acute vertigo / dizziness”	Combining the “essential in acute vertigo / dizziness” score and the “timing & triggers” score
“Superscore for episodic / chronic vertigo / dizziness”	Combiding the “essential in episodic / chronic vertigo / dizziness” score and the “timing & triggers” score
“Education” score	Analogue media (hands-on courses, workshops, national recommendations, practical recommendations) and digital media (smartphone apps, webinars).

A series of scores to reflect key aspects of the diagnostic workup (both history taking and bedside testing) were predefined by the authors (AZ, GM, AAT) and were used to correlate with several epidemiologic aspects including years of professional experience, location of the specialists’ office and reported number of dizzy patients evaluated. These scores were graded based on the extent to which the specialists agreed with a given procedure or the indicated importance of a proposed measure, ranging from e.g. 3 points (very important / fully agreed; 100%) and 2 points (rather important / partially agreed) to 1 point (rather unimportant / partially disagree) and 0 points (not important at all / disagree at all); or if binary 1 points = e.g. agree/used (100%) to 0 points = disagree/not used (0%). Overall scores were derived from the sum of the underlying items and then indexed to 0-100%; or if derived from different scales first indexed and then averaged. Fractional regressions (odds ratios (OR) with 95% confidence intervals (CI)) are reported for indexed scores; binary dependent variables were analyzed using logistic regression (OR with 95% CI). Descriptive statistics report means with standard deviations (±SD), or medians with inter-quartiles (25% to 75%) or counts with percentages (% of non-missing cases); and sample sizes (number of respondents).

Table S2: Predictors for a lacking specific diagnosis in acutely dizzy patients after the first consultation

Table S2: Predictors for a lacking specific diagnosis in acutely dizzy patients after the first consultation			
	**Univariable**		
**Predictor**	**Sample size (n)**	**Odds ratio (95% CI)**	**p-value**
Age			0.003
aged 30-40 years	29	2.03 (1.15-3.59)	0.015
aged 41-50 years	33	1.21 (0.69-2.12)	0.508
aged 51-60 years	28	0.99 (0.58-1.70)	0.967
aged > 60 years	21	[Ref.]	
Gender			
male	71	[Ref.]	
female	40	1.04 (0.72-1.51)	0.835
Years of professional experience	111	0.78 (0.64-0.94)	0.008
Location of specialists' office			
German part of Switzerland	87	[Ref.]	
Latin part of Switzerland	24	1.61 (1.11-2.33)	0.012
Number of dizzy patients seen monthly	111	0.99 (0.98-1.00)	0.085
Availability of Frenzel goggles	111	0.69 (0.33-1.48)	0.346
Score "timing and triggers" (0-100%)	111	0.91 (0.79-1.05)	0.198
Score "ataxia of stance and gait" (0-100%)	111	0.94 (0.84-1.04)	0.204
Score "oculomotor and vestibular signs" (0-100%)	111	0.90 (0.77-1.05)	0.193
Superscore acute dizziness (0-100%)*	111	0.82 (0.66-1.01)	0.065
	**Multivariable**		
**Predictor**	**Sample size (n)**	**Adjusted odds ratio (95% CI)**	**p-value**
Age			0.265
aged 30-40 years	29	2.23 (0.67-7.38)	0.189
aged 41-50 years	33	1.30 (0.52-3.25)	0.575
aged 51-60 years	28	1.09 (0.59-2.00)	0.787
aged > 60 years	21	[Ref.]	
Location of specialists' office			
German part of Switzerland	87	[Ref.]	
Latin part of Switzerland	24	1.54 (1.07-2.23)	0.021
Years of professional experience	111	0.99 (0.65-1.50)	0.970
Number of dizzy patients seen monthly	111	0.99 (0.97-1.00)	0.160
Score "timing and triggers" (0-100%)	111	1.10 (0.88-1.39)	0.401
Score "oculomotor and vestibular signs" (0-100%)	111	0.92 (0.74-1.15)	0.480
Superscore acute dizziness (0-100%)*	111	0.95 (0.61-1.48)	0.809

Abbreviations: CI=confidence interval; Ref=reference. Logistic regressions.

* The "superscore acute dizziness" combines the "essential for acute dizziness" score (assessing gait, Romberg test, head-impulse test, spontaneous nystagmus with fixation and without fixation, testing for gaze-evoked nystagmus, testing for a skew deviation and for hearing loss (using finger rubbing)) and the "timing and triggers" score (asking for the frequency of dizzy episodes, for triggers including specific movements and situations, the duration of dizzy episodes and other, accompanying symptoms)

Table S3: Predictors for a lacking specific diagnosis in patients with episodic/chronic dizziness after the first consultation

Table S3: Predictors for a lacking specific diagnosis in patients with episodic / chronic dizziness after the first consultation			
	**Univariable**		
**Predictor**	**Sample size (n)**	**Odds ratio (95% CI)**	**p-value**
Age			0.015
aged 30-40 years	29	1.98 (1.19-3.31)	0.009
aged 41-50 years	33	1.31 (0.77-2.24)	0.322
aged 51-60 years	28	1.12 (0.65-1.94)	0.678
aged > 60 years	21	[Ref.]	
Gender			
male	71	[Ref.]	
female	40	0.91 (0.64-1.30)	0.606
Years of professional experience	111	0.80 (0.68-0.95)	0.009
Location of specialists' office			
German part of Switzerland	87	[Ref.]	
Latin part of Switzerland	24	1.61 (1.10-2.36)	0.015
Number of dizzy patients seen monthly	111	0.99 (0.97-1.00)	0.052
Score "timing and triggers" (0-100%)	111	0.81 (0.67-0.98)	0.034
Score "oculomotor and vestibular signs" (0-100%)	111	0.80 (0.68-0.94)	0.007
Score "episodic/chronic dizziness" (0-100%)	111	0.74 (0.62-0.88)	<0.001
Superscore episodic/chronic dizziness (0-100%)*	111	0.64 (0.51-0.80)	<0.001
	**Multivariable**		
**Predictor**	**Sample size (n)**	**Adjusted odds ratio (95% CI)**	**p-value**
Age			0.409
aged 30-40 years	29	2.32 (0.71-7.54)	0.162
aged 41-50 years	33	1.47 (0.62-3.50)	0.381
aged 51-60 years	28	1.24 (0.65-2.36)	0.507
aged > 60 years	21	[Ref.]	
Location of specialists' office			
German part of Switzerland	87	[Ref.]	
Latin part of Switzerland	24	1.27 (0.85-1.89)	0.238
Years of professional experience	111	1.00 (0.67-1.51)	0.988
Number of dizzy patients seen monthly	111	0.99 (0.98-1.01)	0.237
Score "timing and triggers" (0-100%)	111	0.96 (0.80-1.15)	0.650
Score "oculomotor and vestibular signs" (0-100%)	111	0.87 (0.70-1.07)	0.193
Score "episodic/chronic dizziness" (0-100%)	111	0.84 (0.69-1.04)	0.105

Abbreviations: CI=confidence interval; Ref=reference. Logistic regressions.

* The "superscore episodic/chronic dizziness" combines the "essential for episodic/chronic dizziness" score (performing provocation maneuvers, the head-impulse test, gait analysis and the Romberg test) and the "timing and triggers" score (asking for the frequency of dizzy episodes, for triggers including specific movements and situations, the duration of dizzy episodes and other, accompanying symptoms)

Table S4: Predictors for referring acutely dizzy patients to a specialist

Table S4: Predictors for referring acutely dizzy patients to another specialist			
	**Univariable**		
**Predictor**	**Sample size (n)**	**Odds ratio (95% CI)**	**p-value**
Age			0.004
aged 30-40 years	29	1.49 (0.79-2.81)	0.222
aged 41-50 years	33	1.14 (0.59-2.19)	0.704
aged 51-60 years	28	0.47 (0.25-0.91)	0.025
aged > 60 years	21	[Ref.]	
Gender			
male	71	[Ref.]	
female	40	1.31 (0.78-2.18)	0.307
Years of professional experience	111	0.78 (0.61-1.00)	0.048
Location of the specialist's working place			
German part of Switzerland	87	[Ref.]	
Latin part of Switzerland	24	2.84 (1.63-4.93)	<0.001
Location of specialist's working place			
Private office on the countryside	9	[Ref.]	
Private office in the agglomeration / in the city	53	2.01 (0.96-4.22)	0.064
Hospital (academic/non-academic)	49	3.53 (1.74-7.18)	<0.001
Number of physicians working in the specialist's location			0.062
1 physician	28	[Ref.]	
2-4 physicians	42	0.59 (0.31-1.11)	0.103
≥ 5 physicians	41	1.04 (0.56-1.96)	0.894
Number of dizzy patients seen monthly	111	0.98 (0.95-1.00)	0.108
Diagnosis remained unclear after initial assessment	111	1.37 (1.22-1.54)	<0.001
Superscore acute dizziness (0-100%)*	111	0.80 (0.56-1.15)	0.227
	**Multivariable**		
**Predictor**	**Sample size (n)**	**Adjusted odds ratio (95% CI)**	**p-value**
Age			0.123
aged 30-40 years	29	0.87 (0.41-1.85)	0.718
aged 41-50 years	33	1.13 (0.58-2.18)	0.726
aged 51-60 years	28	0.56 (0.28-1.10)	0.091
aged > 60 years	21	[Ref.]	
Location of specialist's working place			
German part of Switzerland	87	[Ref.]	
Latin part of Switzerland	24	2.07 (1.27-3.37)	0.004
Number of physicians working in the specialist's location			0.083
1 physician	28	[Ref.]	
2-4 physicians	42	0.57 (0.31-1.04)	0.068
≥ 5 physicians	41	0.46 (0.21-1.01)	0.054
Number of dizzy patients seen monthly	111	0.99 (0.97-1.01)	0.253
Diagnosis remained unclear after initial assessment	111	1.24 (1.08-1.41)	0.002

Abbreviations: CI=confidence interval; Ref=reference. Logistic regressions.

* The “superscore acute dizziness” combines the “essential for acute dizziness” score (assessing gait, Romberg test, head-impulse test, spontaneous nystagmus with fixation and without fixation, testing for gaze-evoked nystagmus, testing for a skew deviation and for hearing loss (using finger rubbing)) and the “timing and triggers” score (asking for the frequency of dizzy episodes, for triggers including specific movements and situations, the duration of dizzy episodes and other, accompanying symptoms)

Table S4: Predictors for referring patients with episodic / chronic vertigo or dizziness to another specialist (n=111)

Table S4: Predictors for referring patients with episodic or chronic vertigo or dizziness to another specialist			
	**Univariable**		
**Predictor**	**Sample size (n)**	**Odds ratio (95% CI)**	**p-value**
Age			0.145
aged 30-40 years	29	1.39 (0.62-3.09)	0.425
aged 41-50 years	33	0.91 (0.41-2.00)	0.805
aged 51-60 years	28	0.66 (0.31-1.41)	0.282
aged > 60 years	21	[Ref.]	
Gender			
male	71	[Ref.]	
female	40	1.18 (0.69-2.04)	0.545
Years of professional experience	111	0.83 (0.62-1.12)	0.222
Location of specialist's working place			
German part of Switzerland	87	[Ref.]	
Latin part of Switzerland	24	2.61 (1.47-4.63)	0.001
Location of specialist's working place			<0.001
on the countryside	9	[Ref.]	
in the agglomeration / in the city	53	2.84 (1.47-5.49)	0.002
in the hospital (academic / non-academic)	49	3.47 (1.84-6.57)	<0.001
Number of physicians working in the PCPs' office			0.020
1 physician	28	[Ref.]	
2-4 physicians	42	0.54 (0.29-1.02)	0.058
≥ 5 physicians	41	1.14 (0.59-2.19)	0.697
Number of dizzy patients seen monthly	111	0.97 (0.95-1.00)	0.045
Diagnosis remained unclear after initial assessment	111	1.20 (1.06-1.35)	0.004
Superscore episodic/chronic dizziness (0-100%)*	111	0.70 (0.49-0.99)	0.044
	**Multivariable**		
**Predictor**	**Sample size (n)**	**Adjusted odds ratio (95% CI)**	**p-value**
Age			0.639
aged 30-40 years	29	1.18 (0.48-2.89)	0.726
aged 41-50 years	33	1.01 (0.44-2.32)	0.974
aged 51-60 years	28	0.81 (0.39-1.69)	0.567
aged > 60 years	21	[Ref.]	
Location of specialist's working place			
German part of Switzerland	87	[Ref.]	
Latin part of Switzerland	24	1.77 (0.92-3.41)	0.086
Location of specialist's working place			0.042
on the countryside	9	[Ref.]	
in the agglomeration / in the city	53	2.65 (1.24-5.67)	0.012
in the hospital (academic / non-academic)	49	2.22 (0.90-5.49)	0.085
Number of physicians working in the PCPs' office			0.078
1 physician	28	[Ref.]	
2-4 physicians	42	0.61 (0.32-1.17)	0.138
≥ 5 physicians	41	1.24 (0.43-3.62)	0.693
Number of dizzy patients seen monthly	111	0.98 (0.96-1.00)	0.117

Diagnosis remained unclear after initial assessment	111	1.15 (1.01-1.31)	0.039
Superscore episodic/chronic dizziness (0-100%)*	111	1.01 (0.69-1.47)	0.965

Abbreviations: CI=confidence interval; Ref=reference. Logistic regressions.

* The "superscore episodic/chronic dizziness" combines the "essential for episodic/chronic dizziness" score (performing provocation maneuvers, the head-impulse test, gait analysis and the Romberg test) and the "timing and triggers" score (asking for the frequency of dizzy episodes, for triggers including specific movements and situations, the duration of dizzy episodes and other, accompanying symptoms).

REFERENCES

1. Newman-Toker DE, Edlow JA (2015) TiTrATE: A Novel, Evidence-Based Approach to Diagnosing Acute Dizziness and Vertigo. Neurol Clin 33:577-599, viii
2. Kattah JC, Talkad AV, Wang DZ, Hsieh YH, Newman-Toker DE (2009) HINTS to diagnose stroke in the acute vestibular syndrome: three-step bedside oculomotor examination more sensitive than early MRI diffusion-weighted imaging. Stroke 40:3504-3510
3. Newman-Toker DE, Kerber KA, Hsieh YH, Pula JH, Omron R, Saber Tehrani AS, Mantokoudis G, Hanley DF, Zee DS, Kattah JC (2013) HINTS outperforms ABCD2 to screen for stroke in acute continuous vertigo and dizziness. Acad Emerg Med 20:986-996
